# Legal medicine implications of a multidisciplinary approach to managing Traumatic Encephalopathy Syndrome in Australia

**DOI:** 10.3389/fneur.2023.1179319

**Published:** 2023-06-30

**Authors:** Peter S. Kim, Roy G. Beran

**Affiliations:** ^1^Australasian College of Legal Medicine, Sydney, NSW, Australia; ^2^South Western Clinical School, University of New South Wales, Sydney, NSW, Australia; ^3^South Western Sydney Area Health Service, Ingham Institute for Medical Research, Liverpool, NSW, Australia; ^4^School of Medicine, Griffith University, Southport, QLD, Australia; ^5^Department of Medical Law, Sechenov Moscow First State University, Moscow, Russia; ^6^Medical School, Western Sydney University, Sydney, NSW, Australia

**Keywords:** Chronic Traumatic Encephalopathy, Traumatic Encephalopathy Syndrome, multidisciplinary team, legal medicine, duty of care, treatment

## Abstract

The medical profession has a fundamental obligation to accurately diagnose and effectively treat a range of diseases and conditions. In the case of Traumatic Encephalopathy Syndrome (TES), where there are no universally accepted clinical diagnostic criteria, a clear clinical diagnosis can pose significant challenges for healthcare providers and for subsequent appropriate management. “Nihilism” or an uncertain working diagnosis is not acceptable in the medical field and deserves further consideration. This paper explores the legal obligations that are placed upon healthcare professionals, both individually and as a part of a multidisciplinary team. This article analyses the responsibilities and expectations of medical professionals in diagnosing and treating complex medical conditions, such as TES. The authors address legal issues that must be considered for an effective operation of integrated medicine to enhance the overall quality of care and improving patient outcomes for those affected with underlying Chronic Traumatic Encephalopathy (CTE).

## Traumatic Encephalopathy Syndrome: a complex and challenging diagnosis

Chronic Traumatic Encephalopathy (CTE) remains a post-mortem diagnosis and, like other tauopathies, relies on post-mortem examination of brain tissue, prepared with tau immunochemical stain (1). The diagnosis is not available antemortem (2). It is strongly associated with exposure to repeated head trauma (RHT), with a cause-and-effect relationship between RHTs and CTE being presumed (3). It remains unconfirmed whether the pathognomonic CTE lesion, as described by McKee and endorsed by the National Institutes of Health (NIH), is responsible for all symptoms observed in patients later found to have CTE pathology at autopsy. The general consensus of the medical and scientific community is unanimous in that there is a cause-effect relationship (3).

Diagnosing CTE, during the life of individual, equating to Traumatic Encephalopathy Syndrome (TES), is important (4, 5). It will enable physicians to offer patients a range of treatment options and to effectively monitor their responses. Numerous antemortem clinical diagnostic tools are available to diagnose TES (6–10) but they lack validity and authority, as there are no universally approved criteria that are unanimously accepted by the medical profession.

A postal survey, conducted on 3,913 former football players who had received compensation from any National Football League (NFL) team, since 1960, revealed that 108 (2.8%) had been diagnosed with CTE (11). The survey demonstrated that physicians were diagnosing CTE, during life, even though it cannot be officially diagnosed ante-mortem. In response to the growing imperative, to diagnose individuals with CTE antemortem, the National Institute of Neurological Disorders and Stroke (NINDS) published diagnostic criteria for TES in 2021 (2). The TES criteria allow the presumptive identification of individuals with a CTE-like syndrome *in-vivo*, to prospectively collect relevant clinical information, on probable CTE patients, to improve the understanding of the natural progression of CTE, develop better investigative approaches and define pre-existing factors causing CTE (2). The criteria have been endorsed for research purposes but not for clinical use (7).

The NINDS expressed the need for caution when trying to apply the diagnostic criteria for TES in clinical practice as these have not been adequately validated to underwrite their widespread application (9). Despite this caution, the authority of the NINDS and the simplicity of the criteria have prompted physicians to ignore the caution and either to adopt the criteria or use them as a guideline to diagnose TES (12). Given the increasing incidence of CTE, particularly among high-risk groups, such as in collision sports (13) and within military settings (6), the implementation of TES criteria, within clinical medicine, would be an invaluable tool to enhance the ability to diagnose and manage TES/CTE in living individuals and possibly mitigate the associated consequences (10).

## Legal obligations of healthcare professionals in providing accurate diagnoses

The duty of physicians, to diagnose and treat patients properly, has existed since ancient times, stemming from the principle of the sanctity of human life (13). The obligation to care for the ill is fundamental in achieving this principle. The concept of physicians practicing beneficence has been propagated since the times of Hippocrates and is considered a cornerstone of the medical profession (14, 15).

In common law, the physicians has a duty of care to his/her patients. In *Roger v Whitaker,* the duty of care was defined as a “*single comprehensive duty covering all the ways in which a physician is called upon to exercise his skill and judgment; it extends to the examination, diagnosis and treatment of the patient and the provision of information in an appropriate case”* (16). The scope of that duty extends to the obligation to properly diagnose and treat diseases (17), accepting that physicians are obliged to keep up-to-date with the latest diagnostic and therapeutic advancements (18).

The policy reason, to hold the medical profession responsible for the obligation to correctly diagnose and treat, was explained in *Paul v Cooke* (19), which was decided under the provisions of *Civil Liability Act 2002 (NSW)* (CLA) (20). In *Cooke*, the patient became hemiplegic after a berry aneurysm, which was missed by previous radiologist, 2 years earlier, ruptured during a coiling procedure. One issue that arose was whether the radiologist should be held liable for failing to diagnose and treat the berry aneurysm. Brereton J said,

“…in a failure to diagnose case, breach of the duty results in a condition not being discovered and treated, so that the condition persists and potentially deteriorates, continuing to cause harm that could otherwise be avoided or becoming more difficult to treat in the future…

the rationale of the duty in connection with diagnosis is to protect the patient from harm caused by illness or injury that can be avoided or alleviated by treatment.

Unlike the duty to warn, its purpose is not truly to enable a patient to make an informed choice about treatment or to submit to what otherwise would be an assault; rather, it is to enable the appropriate treatments to be identified” (21).

While *Cooke* helped define the duty of care, liability did not fall on the previous radiologist as the aim of the CLA is to place the plaintiff in the position (s)he would have been, had the negligence not occurred. In *Cooke,* the delay in diagnosis was considered not to have caused the subsequent arterial rupture but allowed the patient to consider advances in intervention, including coiling of the aneurysm, which was the cause and accepted risk of the procedure, rather than the delay in diagnosis.

Physicians have an obligation to diagnose and treat diseases, including TES, even though CTE can only be definitively diagnosed post-mortem. An analogy is Parkinson’s disease that is a post-mortem neuropathological diagnosis even though Parkinson’s disease is a syndromal diagnosis based on the finding of two of the four cardinal features which include: bradykinesia; rigidity; tremor; and gait instability or disturbance (22, 23) and the syndrome is an ante-mortem clinical diagnosis (24). TES, the presumed antemortem equivalent of CTE, is potentially diagnosable during life, only if there is accepted endorsement of the TES criteria. The law underwrites the obligation for physicians to ensure that appropriate treatment be provided to mitigate or prevent harm that may be caused by his/her illness.

## Legal obligations in the context of multidisciplinary approach to diagnosing and treating Traumatic Encephalopathy Syndrome

The best standards of practice, for managing chronic illnesses, such as Parkinson’s syndrome, due to their wide constellation of clinical presentations and chronicity of the disease, are best met through a multidisciplinary team (25). NINDS defined TES as “the clinical disorder associated with neuropathologically diagnosed Chronic Traumatic Encephalopathy”(3). This definition makes the assumption that there is a specific, identifiable clinical disorder or constellation of symptoms associated with CTE pathology which is well summarized by McKay as a chronic neurodegenerative condition and can present with disorder of “*mood (depression, mood swings, apathy, anxiety, agitation), changes in behavior (impulsivity or aggressive behavior), changes in cognitive functioning (loss of attention and concentration, short-term memory loss, explosivity, poor judgment and decision-making and language difficulties), suicidality, symptoms of motor neuron diseases (MND), Parkinsonism, or post-traumatic stress disorder (PTSD)*” (26). Due to the complex and wide-ranging clinical features that may require the input from multiple health disciplines, to correctly diagnose and manage TES (13), one can argue that it is best managed by an integrated approach, through a multidisciplinary team (MDT).

## Additional legal considerations in managing TES in the context of a multi- disciplinary team

Additional legal considerations need to be entertained when an MDT is involved in the diagnosis and treatment of TES, as opposed to a single health professional. The following issues must be considered:

(1) Privacy issue

Through direct observation, a study examined real-life situations in which there was a breach of confidentiality in a tertiary hospital. Following 7,138 days and 33,157 h of observation, the authors found an estimated ‘Frequency Index’ of one breach per 62.5 h. The most frequent breaches (54.6%) were related to the consultation and/or disclosure of clinical and/or personal data to medical personnel not involved directly in the patient’s clinical care, as well as to individuals who were external to the hospital (27).

A community survey, conducted by the South Australian Health Commission, of 3,037 adults, revealed that 24 respondents confirmed that their health information was released, without their permission/consent, in the past 12 months (28). Unauthorized access and disclosure of health records were shown to be a systemic problem that required addressing.

In Australia, individual health professionals or organizations (such as MDTs) are considered to be Australian Privacy Principle (APP) entities and are obliged to observe the APPs contained in Schedule 2 of the *Privacy Act 1988* (Cth), as well as the equivalent State provisions in each of the states.

The principle 6.1 governs use and disclosure of the patient’s information, which states:

“If an APP entity holds personal information about an individual that was collected for a particular purpose (the primary purpose), the entity must not use or disclose the information for another purpose (the secondary purpose) unless:

(a) the individual has consented to the use or disclosure of the information; or

(b) subclause 6.2 or 6.3 applies in relation to the use or disclosure of the information” (29).

The primary purpose of health practitioners, when collecting patient health information, is for the diagnosis and treatment of the patient’s disease. Sharing that information, with members of the MDT, to fulfill the primary purpose is permitted under the APP, without requiring the patient’s consent. In New South Wales, the law requires disclosure of the identity of all members of the treating team who will have access to the information, unless this is obvious, from the circumstances of any health service provided, as outlined in the *Health Records (Privacy and Access) Act 2002* (NSW), Schedule 1, Principle 4(1)(d).

Effective integrated clinical care requires the sharing of clinical information across a team, from a range of sectors and disciplines. Physicians may assume that adopting an holistic approach to healthcare and sharing relevant information is always in the patients’ best interest, even though patients may not anticipate nor consent to such an approach (24). In *KJ v Wentworth Area Health Service* (30), an oncology patient who was treated by an MDT, at Nepean Hospital, received counseling by a psychologist and a psychiatrist consultation. Later, the patient discovered that her notes relevant to these consultations, which she felt were confidential, were available in the general medical file. The patient successfully filed a complaint with health authorities and the tribunal upheld her argument “*that there was a ‘lack of alignment between the expectations of patients about how their privacy will be respected and a culture of disclosure that exists in the medical community’*” (31).

It would be prudent to disclose to the patient the nature of the MDT, the identity of its various team members and to seek the patient’s consent to share health information among them. Most patients would agree to this arrangement, as they know that a collaborative and collective decision of the MDT would serve their best interests.

(2) Obligation to update the latest medical and scientific developments and to share with the MBT

In *South Eastern Sydney Area Health Service v King* (32), a 13-year-old child with Rhabdomyosarcoma of the paraspinal muscle, including the spinal cord involvement at the level of C7, was treated with intrathecal chemotherapy, pursuant to the IRS-II [Second Intergroup Rhabdomyosarcoma Study which was superseded with updated IRS III protocol (33)]. The treating physician who was part of the Children’s Tumor Clinic Group of the Prince of Wales Hospital, continued to treat the 13-year-old with an outdated chemotherapy protocol which led to permanent paralysis of the lower limb. The treating physician was unaware of the recent amendment to the protocol, which was modified to prevent the injury of the type the patient suffered. The experts stated that if the treating physician had been aware of the 1987 changes to the protocol on which he had relied, he would have acted unreasonably if he had not followed the revised recommendations made by the amendment.

The court held that the treating physician had an obligation to update his/her knowledge individually, and within the group, which existed as an MDT within the public hospital, with an ongoing obligation to update and share such knowledge. It was found that Dr. White, the pediatric oncologist, who was a member of both MDT and the IRS Group, normally circulated material that he received, relating to such oncology, as part of his usual communication with the pediatric oncology team, but failed to do so on this occasion. As a result, the hospital was found to be vicariously liable for the negligence associated with this failure to share the information with the group, consistent with a systemic failure in communication of important information within the MDT and which was considered to have caused the paralysis.

Individual members of the MDT have an obligation to stay current with the latest medical and scientific developments related to the treatments they are offering. As a group, they also have an additional obligation to share any new information with the other members of the MDT, especially if the treatment has the potential to cause harm to the patient.

(3) Deficient medical record keeping

In *Young v Central Australian Aboriginal Congress* (34), a young Aboriginal man presented to an Aboriginal medical service, which represented a multidisciplinary primary health clinic, complaining of crushing chest pain. The primary care physician referred the patient to a cardiologist but the patient failed to keep the appointment. After several months, the patient died from a heart attack. He had visited the same clinic, on several occasions, before his death but the cardiological problem was never pursued and he did not attend the cardiology consultation nor undergo associated investigations. It was found that an administrative system had existed, at the clinic, to follow up serious medical cases, but it failed, on this occasion (35). The GP was not found to be negligent as he did not have a system, within his own practice, to follow up patients referred for treatment (36). The negligence rested on the physician, in charge of the clinic, whose responsibility it was to follow up missed appointments (37).

In the context of TES being managed in an MDT, it is essential to have a system of timely and accurate medical record-keeping so that all members can contribute to the management of the patient. It is also important to have a designated treating physicians who should regularly review individual cases to ensure that all reports, recommendations and recalls are actioned and followed up in a timely manner.

(4) Organizational liability

In *South Eastern Sydney Area Health Service v King* (27), a lawsuit in negligence was brought against the members of the MDT, the Children’s Tumor Clinic Group operating in the Prince of Wales Hospital. This group consisted of pediatric oncologists, pediatric surgeons and pediatric pathologists who regularly held multidisciplinary sessions, to discuss relevant tumor protocols and review available information. Despite this, Dr. White, a member of the group, had knowledge about the dangers of chemotherapy cocktails, used to treat the patient, but failed to share this information with other members in the group, including the treating physicians. The court held that Dr. White had a duty to pass on information within the MDT (38). Dr. White’s inaction was found to be negligent, and as a result, the hospital was found to be vicariously liable and responsible for $7 million in damages. It is essential, for all members of an MDT, to actively share and update knowledge with the rest of the team, to obviate negligence and ensure the best practice for the patient.

The MDT is generally not a legal entity that can be the subject of litigation. As noted in *King*, the organization that arranges (and usually employs) the members of the MDT may be vicariously liable, as is the situation within a public or private hospital which either employs or refers patients to members of the team, thereby establishing the team’s credibility, for the negligence that might ensue, consequent to the actions or inactions of the members of that MDT.

(5) Limitations

There are several limitations to this review as follows:

Firstly, the descriptions of statutory and case law provided are specific to the Australian jurisdiction and cannot be generalized to other jurisdictions.

Secondly, the authors should caution readers that they are advocating against the NINDS’ expressed need for caution in adopting TES diagnostic criteria in the clinical setting.

Thirdly, the authors acknowledge that validating clinical criteria in other neurological conditions, especially those without genetic causes, can take several years, if not decades. It is likely to take the same or even longer to formalize and create a consensus on possible probability-based TSE criteria. The authors advocate for an alternate approach to diagnosis and intervention, and only time will confirm or refute the approach adopted.

Fourthly, recent neuropathological investigations have shown that TES may extend beyond the concept of CTE, as different populations, not just those in contact sports, exhibit various types of lesions in the context of TBI as well as different types of CTE incidence and distribution (39-41). This makes reaching a possible consensus on CTE-TES particularly challenging at present.

Lastly, there is currently no case law that can be used to better contextualize the legal medicine aspects of TES/CTE.

## Conclusion

The MDT is considered to be the appropriate or preferred model of care for the management of TES, given its complex and chronic clinical presentation. Medical professionals have an obligation to diagnose and treat TES, assuming that there is a respected body of professional peers who accept and adopt the NINDS criteria, despite the warning identifying the potential lack of validity. The MDT, as a consensus body, also has additional legal obligations to respect administrative commitments which include: privacy; communication of medical advances; medical record-keeping; and organizational liability.

The authors acknowledge that there has been criticism of adopting TES diagnostic criteria as a clinical tool. One critique is that the criteria are not validated clinical criteria, and like in other neurological conditions, it will take significant time to formalize and create a consensus. An authoritative or confident clinical diagnosis is not a prerequisite for patients to receive treatment for their symptoms. There is also a risk of falsely diagnosing TES due to the inherent uncertainty of the clinical diagnostic criteria.

The provision of treatment for TES, such as attracting support groups and arranging cohesive multidisciplinary care, is only possible with an authoritative and confident diagnosis. The authors note the caveat of needing the support of a respected body of peers, as defined in the CLA, section 5O. In the effective management of TES, diagnostic *nihilism* is no longer acceptable, and denying the applicability of the TES criteria may leave the treating clinician vulnerable.

## Data availability statement

The original contributions presented in the study are included in the article/supplementary material, further inquiries can be directed to the corresponding author.

## Author contributions

All authors listed have made a substantial, direct, and intellectual contribution to the work and approved it for publication.

## Conflict of interest

The authors declare that the research was conducted in the absence of any commercial or financial relationships that could be construed as a potential conflict of interest.

## Publisher’s note

All claims expressed in this article are solely those of the authors and do not necessarily represent those of their affiliated organizations, or those of the publisher, the editors and the reviewers. Any product that may be evaluated in this article, or claim that may be made by its manufacturer, is not guaranteed or endorsed by the publisher.
